# Medullary allotransplant in acute myeloblastic leukemia in a child

**Published:** 2014-09-25

**Authors:** V Buga Corbu, R Glűck, C Arion

**Affiliations:** * "Carol Davila" University of Medicine and Pharmacy, Bucharest; ** “I. C. Fundeni" Pediatrics Clinic, “Carol Davila" University of Medicine and Pharmacy, Bucharest

**Keywords:** acute myeloblastic leukemia, child, allo-HSCT

## Abstract

Abstract

Although acute myeloblastic leukemia (AML) is more resistant to chemotherapy than acute lymphoblastic leukemia (ALL), significant progresses have been achieved over the last 20 years with an improvement in the long-term survival up to 50-60%. This may be attributed to the intensification of chemotherapy, including the increased use of stem-cell transplantation (HSCT) in well-defined subgroups. Allo-HSCT represents an extremely effective alternative in pediatric AML treatment panel, but its efficiency is limited both by the toxic effects and by the difficulty of finding a matched HLA donor.

 The application of the hematopoietic stem cells transplantation (HSCT) [**5**] as a therapeutic alternative in acute myeloblastic leukemia (AML) in a child has been a subject of debate from the end of the ‘70s and it currently remains a major issue, just like the perfect moment for the performance of HSCT – the first complete remission (CR1), the second complete remission (CR2) or refractory AML. 

 All the clinical studies indicate the allo-HSCT superiority compared with the auto-HSCT as far as the survival without any signs of sickness is concerned. A systematic review and a meta-analysis done on 6 clinical trials compare the allo-HSCT performed from a family donor with the auto-HSCT. As a result of the analysis of 1486 pediatric patients, the HSCT superiority from a matched sibling donor can be observed. 

 According to the current evidence, HSCT [**1,8**] is indicated in CR1 only in patients in the high-risk group (HR); there are studies that demonstrate the allo-HSCT (matched sibling donor) superiority vs. chemotherapy, and other studies that prove that there is no statistically significant difference. 

 Recent evidence from the group in St Jude Children Hospital, Memphis, U.S.A. indicates a radical unfavorable prognosis in children with AML-M7 in whom HSCT was not applied. 

 The American Children cancer Group (CCG), the French group Leucemie Aigue Myeloide Enfant (AMLE), the Italian group Associazione Italiana Ematologia e Oncologia Pediatrica (AIEOP) report the significant superiority of HSCT in CR1 for the HR group in comparison with chemotherapy. 

 The BFM group and Val’Hebron Hospital in Barcelona concluded that long-term toxicity, secondary to HSCT in CR1 for the HR group is more frequent and higher than the case in which chemotherapy is applied. Moreover, other complications have an incidence rate 3-4 times higher in case of transplant: optical atrophy, osteonecrosis at the level of the big joints in fingers and toes, bronchiolitis obliterans, convulsive crises, etc. Significant differences have been remarked in the endocrine parameters: post-transplant gonadotropic hypogonadism has been diagnosed 10 times more frequently than after chemotherapy. 

 Literature data are scarcer as far as HSCT role in CR2 is concerned. Most of the experts prefer allo-HSCT as a complete post remission therapy, especially in case of a total relapse (< 1 year since the diagnosis). 

**Table 1:**
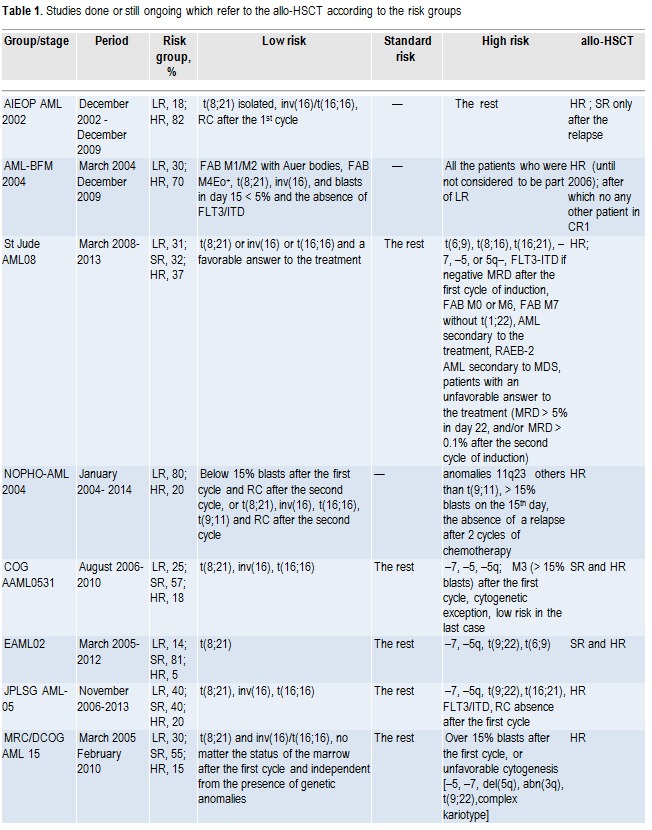
Studies done or still ongoing which refer to the allo-HSCT according to the risk groups

FAB = French-American, British; RAEB, refractory anemia with blasts excess, NOPHO, Nordic Society of Pediatric 

Hematology and Oncology; MRC, Medical Research Council; DCOG, Dutch Childhood Oncology Group. 

Selection of donor 

The donor and the patient are undergoing a HLA class I and II typification by using molecular techniques. It is impossible to adopt some rigorous compatibility criteria, the identification of at least 9 out of 10 alleles, HLA-A,-B,-C,-DRB1, DQB1, being necessary. Before testing the histocompatibility, the infectious screening of the possible donor (HIV, HTLV, HVB, HCV, CMV, EBV, HAV, and serology for syphilis and toxoplasmosis) is necessary, together with the harvesting of the usual biological samples, cardio-pulmonary X-ray and electrocardiogram. 

The persons who are considered potential donors are the following: a relative, with a similar HLA genotype or which differs due to a HLA antigen (brother or sister) non-relative person, identical from the point of view of HLA, or who differs only due to HLA antigen. 

Preservation/ Sampling procedure

The bone marrow harvesting procedure is done while under general or epidural anesthesia. The stem cells quantity which was harvested is expressed as a number of nucleate/kgc cells (the optimum number is minimally 2-5 x10^8, which corresponds to a quantity of 10-15 ml medullary blood/kgc). The bone marrow is harvested in anticoagulant blood bags (heparin 5U/mL marrow), which must be periodically stirred in order to avoid clots formation. Afterwards a leukocyte count is done, which evaluates with a bigger precision the volume of the harvested bone marrow [**6,7**].

The separation of the cells in the bone marrow presupposes the filtration of the bone marrow in order to separate the erythrocytes from leukocytes. 

The Maphosphamide purification procedure 

 It is done only if the number of nucleated cells is minimally 2x10^8/kgc, if not, a new harvesting procedure is imposed. A minimum volume of nucleated cells of 0,5x108/kg is separated and stored as backup for 30 days from the reinfusion of the purified cells. 

 A standard dosage of Maphosphamide of 100µg/20x10^6 nucleated cells is used. 

 The cryopreservation of the purified bone marrow 

 The cellular product obtained after the purification will be frozen in order to be preserved by using a special procedure. 

The evaluation of the viability and the clonogenic potential 

 Is applied both for the purified bone marrow and for the backup, by using vital colors and cellular cultures from the harvested product. 

Establishing the venous access 

 The positioning of a central venous catheter (CVC) in the form of a tunnel, with one or two ways in patients who are eligible for a hematopoietic stem cells transplant is compulsory. 

CVCs in the form of a tunnel – Broviac or Leonard -, which are partially implantable, with a partially subcutaneous trajectory and are partially external, provided at the end with an adapter which can be connected to the medication administration device, will be used. These catheters are known for their possibility of being kept for a long period of time. 

 Due to the high risk of infection, the CVC manipulation has rigorous asepsis rules. The CVC Heparinization prevents the appearance of thromboembolic events but also of infections because the fibrin deposits and the thrombi on the catheters can serve as implantation site for the intraluminal bacterial colonization. The heparin volume of 50U/mL that is used is of 3mL, no matter the caliber. The periodical replacement of the cap is necessary to prevent the infectious risk and it is imposed weekly, during all the period CVC is maintained, if there are signs of its alteration or during the contact with unsterile materials. 

 Blood sampling: after the opening of the catheter, 5ml of blood, which are usually eliminated, are suctioned, followed by the suction of the proper volume for the necessary biological samples. After the blood sampling, CVC is always cleaned with 10ml of SF. The same procedure is applied if before the blood sampling, a hydration solution was administered. However, if a parenteral nutrition solution was administered, the volume of blood eliminated will be of 8 ml and will be preceded by a 10ml SF cleaning.

 The use of CVC with AML in children facilitates the therapeutic maneuvers and the comfort of the patients, but the procedure can lead to different complications: some at the moment of insertion (hemorrhage, blood vessels perforation, hematomas, pneumothorax, hemothorax, hydrothorax, pulmonary atelectasis, arrhythmias, lesions of the brachial plexus, anesthetic risk), local complications (phlebitis, necroses at the CVC exit point, cutaneous ulcers), mechanic complications (faulty positioning, spontaneous displacement, occlusion, rupture), systemic complications (infection, thrombosis, pulmonary embolism, rejection reactions, thrombocytopenia induced by heparin). 

 The conditioning regimen in AML has three main objectives: “creating space", immunosuppression and disease eradication. The use of potentially high immunosuppressant drugs has as a main goal the assurance of the grafting without rejection, a condition that is not compulsory in the case of auto-HSCT. Due to the fact the total body irradiation (TBI) did not prove to have a favorable impact on survival rate without any disease events in the first total remission in children, the regimens used by most of the protocols do not provide TBI as a conditioning regimen in AML, due to late sequelae. Most of the working groups recommend a regimen based on Busulfan, Cyclophosphamide and most of the times Melphalan. 

**Fig. 1:**
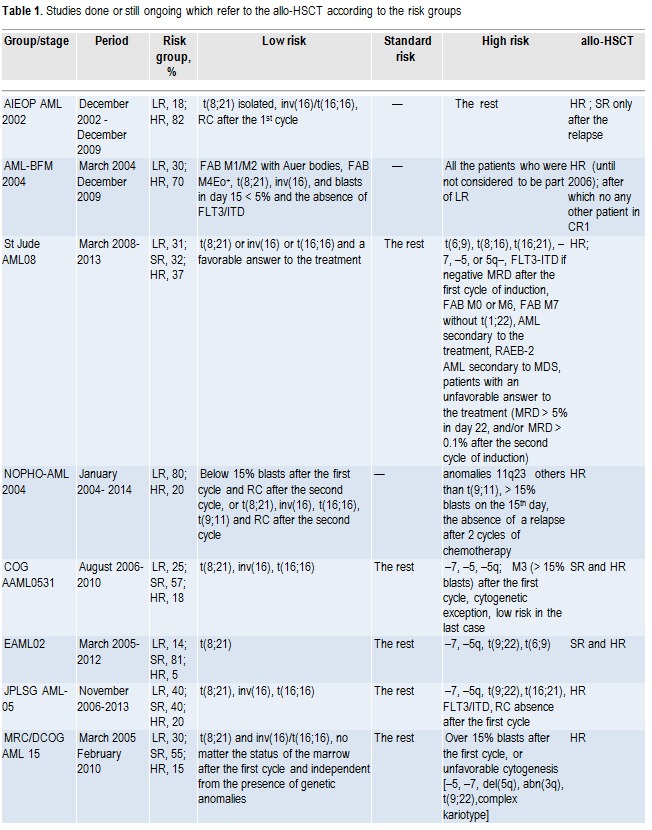
The AML conditioning regimen in children

Busulfan 16mg/kg/day = 4mg/kg x 4 administrations, po, 4 days, day -8, -7, -6, -5.

 In obese patients, it is recommended that the calculation of the ideal weight and the careful monitoring of busulfan are done, the optimum values being between 500 and 700ng/ml. The monitoring of the plasmatic value of Busulfan is recommended to all the patients to whom the administration is done orally. The pharmacokinetics presupposes the harvesting of blood samples before the administration, at 1, 2, 4, 6 hours from the first administration. 

 The anticonvulsant treatment is recommended to all the patients, especially to the ones who are over 6 years old; valproic acid/ carbamazepine/ diazepam/ nitrazepam are administered in usual doses. 

 Cy=Cyclophosphamide 60mg/kg/day, pev 1 hour with 250ml/mp serum glucose, 2 days, day -4, -3.

The cyclophosphamide is an alkylating agent that implies phenomena of acute toxicity (myelosuppression, hemorrhagic cystitis, nausea, vomiting, SIADH alopecia, immunosuppression, sterility) and chronic (gonadal toxicity). In obese patients, the calculation of the ideal weight is necessary. With at least 12 hours before and at least 24 hours after the administration of Cy, hydration with salt and water solutions is recommended in order to prevent metabolic imbalances and hemorrhagic cystitis, according to the following scheme: 

 Hydration: 3000ml/mp/day (in children with a body weight of below 10 kg: 150ml/kg/day).

 Uromitexan: at least 150% of the Cy daily dose; This agent acts locally at the level of the urinary tract by inactivating the urotoxic metabolite of cyclophosphamide, acrolein. 

L-PAM=Melphalan:140mg/kg/day, pev ui SF duration 20 min, 1 day, day-2

The preparation of the patient presupposes: 

 - Prehydration – is initiated in order to assure a corresponding diuresis and to maintain the urinary PH to >6,5.

 - Premedication – presupposes the administration of the antipyretic, antihistaminic, antiemetic and diuretic treatment. 

 The administration of the marrow perfusion 

 After the defrosting of the M.O. bags near the bed of the patient, in a water bath at 37 degrees Celsius, the suspension of cells is administered through CVC in a rhythm of 10-15mL/minute, under the strict surveillance of a doctor. 

 The main cause of failure in allo-HSCT remains a engraft disease against the host (GvHD), a situation in which the donated cells recognize the cells of the receiver as non-self antigen, which has as a consequence the appearance of cutaneous, gastro-intestinal and hepatic manifestations with various degrees of severity, from the Ist to the IVth degree. The main factors which influence the GvHD development are connected to the donor-host relationship (HLA incompatibility), an incompatibility regarding sex, age, the stem cells source (the marrow, peripheral blood, umbilical cord) and the conditioning regimen used. If in the past, the intravenous administration of immunoglobulins was a normal immunomodulation practice in allo-HSCT patients, this method was abandoned due to the high price but also due to the development of other GvHD prevention techniques. In order to reduce the GvHD incidence, techniques of T-lymphocytes removal from the blood of the donor have been developed, these being the main cells responsible for the graft-versus-host-disease. Although the patients who benefit from a graft with T-lymphocytes depletion present lower GvHD rates, higher rates of graft rejection are registered, as well as cytomegalovirus (CMV) infections, invasive fungal infections and infections with Epstein-Barr (EBV) virus, associated in the last case with post-transplant lymphoproliferative disease. 

 Although rarely met in children compared to the adults, chronic GvHD – limited or extensive – develops at >100 days post-transplant, also appearing starting from 40 days post-transplant. Chronic GvHD has the aspect of an autoimmune disease of the conjunctive test (for example, sclerodermia or systemic lupus erythematosus), being associated with deficiencies of the cellular and humoral immunity, deficiencies of the macrophages, PMN chemotaxis disorders, altered answer to vaccinations and severe mucositis. The risk factors for chronic GvHD development are age, allogenic transplant (especially from an unrelated donor or from a related donor who is partially compatible) [**2,4**] and the history of acute GvHD. Although total immunoglobulins are normal or even raised, the level of IgA, IgG and subclasses IgG are decreased and the opsonization and the reticulum-endothelial function are altered. The restoration of immunity after chronic GvHD needs many years, being a process that takes place gradually. 

The prophylaxis recommendations of the graft-versus-host-disease (GvHD) according to the AIEOP study group are the following: 

 Transplant from a family donor who is HLA-identical: 

 • Cyclosporine-A: 2 mg/Kg/day i.v., in 2 administrations, from the 3rd day until approximately in day +20 - +25 post-transplant. It is orally administered successively in doses of 4 – 6 mg/Kg/day. After 6-8 months post-transplant, the immunosuppressive treatment can be gradually decreased to 1 year post-transplant. The Cyclosporine A plasmatic level must be monitored and must be maintained between 100 and 250 ng/mL. 

 • Methotrexate administered on short-term: 15 mg/m2 i.v. in 30 minutes from day +1; 10 mg/mp i.v. in 30 minutes in day +3 and +6 post-transplant.

In the case of unrelated voluntary donor transplant, the following are recommended: 

• Cyclosporine-A: administered according to the same dosage as in the case of a family donor who is HLA identical

• Methotrexate administered on short-term: 15 mg/m2 i.v. in 30 minutes from day +1; 10 mg/mp i.v. in 30 minutes from day +3, +6, +11 post-transplant.

 • ATG: Fresenius 5 -10 mg/Kg/die i.v. or Genzyme 2 – 3 mg/Kg/day, in vep 6 –

 8 hours, from the 5th day to the -3 day. Premedication which includes methylprednisolone and antihistamines. 
